# Fertility knowledge, contraceptive use and unintentional pregnancy in 29 African countries: a cross-sectional study

**DOI:** 10.1007/s00038-020-01356-9

**Published:** 2020-04-09

**Authors:** Ayodeji Emmanuel Iyanda, Barbara J. Dinkins, Tolulope Osayomi, Temitope Joshua Adeusi, Yongmei Lu, Joseph R. Oppong

**Affiliations:** 1 Department of Geography, Texas State University, San Marcos, TX USA; 2 Department of Criminal Justice, Texas State University, San Marcos, TX USA; 3 Department of Geography, University of Ibadan, Ibadan, Nigeria; 4 Center for Gender and Development, Ado-Ekiti, Ekiti State Nigeria; 5 Department of Geography and Environment, University of North Texas, Denton, TX USA

**Keywords:** Adolescence, Reproductive health, Knowledge of ovulation, Fertility awareness, Contraception, Unintentional pregnancy, Medical geography, Africa

## Abstract

**Objectives:**

We examined the association between incorrect knowledge of ovulation and unintentional pregnancy and child among young women in sub-Saharan Africa countries.

**Methods:**

Using Pearson’s Chi-square, *t* test, multiple logistic regression, and likelihood ratio test, we analyzed Demographic and Health Survey data (2008–2017) of 169,939 young women (15–24 year).

**Results:**

The range of prevalence of incorrect knowledge of ovulation was 51% in Comoros and 89.6% in Sao Tome and Principe, while unintentional pregnancy ranged between 9.4% in the Republic of Benin and 59.6% in Namibia. The multivariate result indicates a strong association between incorrect knowledge of ovulation and unintentional pregnancy (OR = 1.17; *p* < 0.05) and unintentional child (OR = 1.15; *p* < 0.05).

**Conclusions:**

Adolescent women (15–19) generally have poor knowledge of ovulation and are more likely to report an unintentional pregnancy/child than women between ages 20–24. To reduce the burden of unintentional child/pregnancy in Africa, fertility knowledge should not only be improved on but must consider the sociocultural context of women in different countries that might affect the adoption of such intervention programs. Pragmatic efforts, such as building community support for young women to discuss and share their experiences with professionals and educate them on fertility and sexuality, are essential.

## Introduction

About 16 million adolescents (15 to 19 years) and nearly one million girls less than 15 years of age give birth every year, worldwide (World Health Organization 2018). Although not all are unintentional and unwanted, adolescent pregnancy remains a social as well as a public health concern (Sedgh et al. 2015). The current gap in unintended pregnancy among countries in Africa is 10–54% (Ameyaw et al. 2019), higher than the previous 20–40% (Hubacher et al. 2008). Teenage pregnancy is highest in African countries, and “teenage marriages are a larger factor than unwanted conceptions in many of the countries with the most teen pregnancies” (Burton 2017). According to the Centers for Disease Control and Prevention (CDC), “unintended or unwanted pregnancy is a core concept for understanding the fertility of populations” (CDC 2015). However, many reproductive health research on adolescents and youth tend to overlook the importance of understanding the fertility window or ovulatory phase with an unintentional pregnancy and tend to focus on the use of contraception (Morris and Rushwan 2015; Kost et al. 2017).

Fertility awareness is the knowledge about the possibility to conceive during the menstrual cycle (Hampton and Mazza 2015), identifying the associated risks of sexual behavior, and decision making about childbearing among sexually active women. The lack of knowledge of fertility period in the absence of or misuse of contraceptives could lead to multiple undesirable health outcomes such as unintentional or unplanned pregnancy in marriage, and unsafe abortion among unmarried and vulnerable populations (e.g., adolescents) (Garenne and Zwang 2006; Thijssen et al. 2014; Hampton and Mazza 2015). The fertility awareness-based method has been offered as an alternative method of family planning (Pallone and Bergus 2009). Although the use of contraception may be considered as a comprehensive strategy for family planning, its effectiveness is being questioned as the rate of unintentional pregnancy is increasing, particularly in developing countries due to failure (Polis et al. 2016; Ameyaw et al. 2019; Srinivasan and White 2019).

Thus, adequate knowledge of ovulatory phase or fertility awareness may assist in reducing the rate of unintentional pregnancy, especially in Sub-Saharan Africa (SSA) where access to contraception is low (see Chandra-Mouli et al. 2014; Blackstone et al. 2017; Kalamar et al. 2018; Radovich et al. 2018; Ba et al. 2019).

The existing literature has shown the nexus between poor knowledge of reproductive health and poor reproductive health outcomes among young people, mostly in developing countries (Sarkar et al. 2015; Chung et al. 2018). Notably, the role of marriage in unplanned or unintentional pregnancy is not yet clear (Okonofua 1995; Fite et al. 2018). The prevention of unintended pregnancy among adolescents and youths, therefore, requires adequate information about reproductive health, sexual behaviors, and associated implications. Thus, reducing the prevalence of unintentional pregnancy should be a priority among policy-makers and public health administrators because of the high economic, social, and health costs to adolescent parents and their families, which include maternal and child mortality, obstetric fistula, and symptoms of poor mental health (Solomon-Fears and Ronquillo 2000).

Based on this forgoing, this paper argues that unintentional pregnancy occurs as a result of the interplay of factors such as inadequate use of contraception or a complete lack of access to it and also inadequate knowledge of the fertility period. It is pertinent, therefore, to examine the interrelationship between knowledge of ovulation and contraception among young women between the ages of 15 and 24 years in 29 African countries. Hopefully, these empirical findings will reiterate the need to intensify sexual education and preventive measures targeting at-risk women and ultimately reduce the burden of unintentional pregnancy in developing countries, especially those in SSA.

## Methods

### Data description

This study used the standard version of Demographic and Health Surveys (DHS), a population-based data available for 29 African countries. The DHS data are available in a publicly available file, free, and accessible based on permission. International organizations that provided financial and technical supports include the United States Agency International Development (USAID), the United Kingdom Department for International Development (DFID), and the United Nations Population Fund (UNFPA). Table 1 contains the list of countries, survey year, and the sample size contribution to the pooled sample from each country. The sample frame used for this study was 169,939 young women between the ages of 15 and 24 years. Because this study only focused on a fertility intention (whether current or previous pregnancy was wanted or unwanted/mistimed/unintended which were operationalized as unintentional pregnancy for consistency), the total initial sample frame was reduced and thus differs for various analyses.

**Table 1:** Country profile and data distribution among women age 15–24 in 29 Sub-saharan African countries between 2008 and 2017

DHS country (year)	Sample size	Share (%)	Ovulatory knowledge		Contraceptive use		Pregnancy desire	
			No (%)	Yes (%)	Yes (%)	No (%)	Yes (%)	No (%)
			(*n* = 169,011)	(*n* = 60,725)	(*n* = 9820)	(*n* = 351)	(*n* = 5640)	(*n* = 736)
Nigeria (2013)	14,459	8.52	79.7	20.3	86.1	13.9	88.5	11.5
Rwanda (2014–2015)	5252	3.09	79	21	80	20	62.2	37.8
Ghana (2008)	1906	1.12	66	34	83.7	16.3	57.8	42.2
Zimbabwe (2010–2011)	3795	2.24	84.8	15.2	63.8	36.2	66.8	33.2
Malawi (2015–2016)	10,367	6.11	82.9	17.1	61.8	38.2	59.8	40.2
CR Congo (2013–2014)	7661	4.51	57.5	42.5	84	16	67.7	32.3
Kenya (2014)	11,483	6.76	76.6	23.4	68.3	31.7	62.6	37.4
Zambia (2013–2014)	6726	3.96	78.5	21.5	71.3	28.7	57.1	42.9
Togo (2013–2014)	3337	1.97	57.2	42.8	80.9	19.1	69.3	30.7
Namibia (2013)	3577	2.11	86.1	13.9	53.4	46.6	40.4	59.6
Sierra Leon (2013)	6739	3.97	69.7	30.3	75.2	24.8	81.6	18.4
Angola (2015–2016	6423	3.78	88.6	11.4	69.9	30.1	76.2	23.8
Burkina Faso (2010)	6592	3.88	63.4	36.6	88.5	11.5	62.5	37.5
Benin (2011–2012)	5742	3.38	57.3	42.7	85.6	14.4	90.6	9.4
Burundi (2016–2017)	7218	4.25	78.2	21.8	86.1	13.9	78.2	21.8
Cameroon (2011)	6708	3.95	68.9	31.1	86.5	13.5	68	32
Comoros (2010)	2282	1.34	51	49	76.6	23.4	70.9	29.1
Ethiopia (2016)	6401	3.77	76.7	23.3	87.3	12.7	65.1	34.9
Gabon (2012)	3407	2.01	55.4	44.6	81.1	18.9	82.3	17.7
Gambia (2013)	4564	2.69	76.9	23.1	67.8	32.2	53.7	46.3
Guinea (2012)	3808	2.13	76.7	23.3	95.7	4.3	84.8	15.2
Lesotho (2014)	2842	1.67	73.8	26.2	90.1	9.9	79.6	20.4
Liberia (2013)	3499	2.06	88.5	11.5	56.9	43.1	46.9	53.1
Madagascar (2008–2009)	6935	4.08	52.7	47.3	78.5	21.5	61.8	38.2
Sao Tome and Principe (2008–2009)	987	0.58	89.6	10.4	73.5	26.5	87.8	12.2
Tanzania (2015–2016)	5399	3.18	82.2	17.8	70.1	29.9	51.9	48.1
Uganda (2016)	8058	4.75	79.7	20.3	76.3	23.7	65.9	34.1
Cote d’Ivoire (2011–2012)	3984	2.35	69.6	30.4	75.3	24.7	57.1	42.9
Chad (2014–2015)	6884	4.05	73.7	26.3	80.2	19.8	69.6	30.4

DHS surveys have large sample sizes, usually between 5000 and 30,000 households, depending on the site’s (country) sampling frame. Most of the respondents came from the Enumeration Areas (EAs) from the most recently completed population census (Demographic and Health Survey Program 2012). The survey usually occurred every 5 years for most participating countries. In addition, the sampling method used in the DHS survey was based on a stratified sampling technique which subdivided administrative units into different clusters and sampled households from these clusters.

### Measures

The primary reproductive health outcomes measured in this study were the prevalence of unwanted pregnancy and unintentional child(ren), based on the questions on fertility intention. There were three responses to each question regarding last child or current pregnancy: “wanted then,” “wanted later,” or “not wanted.” Responses indicating “wanted then” in both questions were recoded as “0 = wanted,” while either question having the response of “wanted later” or “not wanted” was coded as “1 = Unintended.”

The primary explanatory variable of interest was knowledge of the ovulatory phase. Question on knowledge of ovulation phase was elicited from participants and has seven-level responses (1 = During her period, 2 = After the period ended, 3 = Middle of the cycle, 4 = Before period begins, 5 = at any time, 6 = others, 8 = Don’t know). We recoded responses “1, 2, 4, 5, 6, and 8” to “1” which indicates incorrect knowledge and “3” to zero as the correct knowledge. Contraception was used in two forms: pattern of use and methods of use. “Pattern of use” was used in the bivariate analysis, while “methods of use” was used in the multivariate analysis as a control variable. Women who used any method were coded as users (0), and those who reported not using any method were coded as nonusers (1).

Other variables include women’s age, educational attainment, marital status, fertility status, economic status (calculated from wealth index dichotomized as poor and rich), and place of residence. Age was treated as continuous and as a binary variable (“15–19” and “20–24”). Marital status was classified as currently in a relationship, never in a union, or not currently in a union, dummy-coded when necessary. To measure fertility status or parity, the number of children ever born alive was classified as 1, 2, 3, and ≥ 4. We also adjusted for women’s education using the reported highest educational attainment (0 = none, 1 = primary, 2 = secondary, and 3 = higher).

### Analysis techniques

Pearson’s Chi-square technique was used to determine the bivariate association between the binary outcome variable (pregnancy intention) and categorical explanatory variables at the 95% significant level. We also used *t*-test to evaluate the mean difference in age (measured as a continuous variable) between (1) women who correctly identified ovulation phase as the middle of the menstrual cycle and those who did not and (2) those who wanted pregnancy and those who did not intend to conceive their most recent child.

Two types of multivariate logistic regression analyses were performed—main and stratified models. The primary model analyzed aggregated data for two fertility intention outcomes: unintentional pregnancy and child, resulting in four models. The unintentional pregnancy model did not include contraception variables because all women who were currently pregnant were coded as nonusers. Due to the large effect of marital status, we stratified the primary model by marital status, that is, into currently married, previously married, and never married (single). All models included: women’s age (continuous), marital status, educational attainment, fertility, and place of residence. To determine model improvement and fitness in the binary logistic regression, likelihood ratio tests (LR-test) were estimated. We reported the result of the logistic regression based on the odds ratios (ORs) at an acceptable 0.05 alpha level in STATA v12 and SPSS v20.

## Results

### Descriptive and bivariate results

Table 1 presents the distribution of knowledge of ovulation, contraceptive use, and pregnancy desire among women. Of those with a child in the past three to 5 years, 67.63% did not plan their most recent child. The prevalence of incorrect knowledge of ovulation ranged between 51% in the Islands of Comoros and 89.6% in the Islands of Sao Tome and Principe. Table 1 indicates that Namibia had the highest rate of unintended pregnancy of 59.6%, and the Benin Republic had the lowest rate of 9.4%. To check our result for consistency, separate analyses were conducted for women in 15–19 and 20–24 age categories by country of residence. Chi-square confirmed significant difference in knowledge of ovulation for the two groups ($\chi^{2}_{(29)}$ = 5455.26, *p* < 0.001 and $\chi^{2}_{(29)}$ = 5172, *p* < 0.001, respectively (Table not shown).

For the knowledge of the ovulation phase, only 24.98%, including those pregnant women in the sample and 23.5% who were not pregnant, had a correct understanding of the ovulation phase. The mean age of women in our pooled data was 19.18 years old (SD, 2.82). In the pooled data, 57.04% of women were never married, 39.09% were currently married, and 3.87% were previously but not currently in a relationship. In terms of parity, 58.04% of the pooled sample of women aged 15–24 had no children, 22.52% had one child, 12.44% had two children, 5.1% had three children, and 1.9% had four or more children. About one-fifth of women (18.83%) had no education, and the percentage of women that had primary, secondary, and higher education is 35.05%, 42.91%, and 3.21%, respectively. Chi-square test shows significant associations between knowledge of ovulation and all other variables with a *p*-value of less than 0.05 (Table 1).

**Fig. 1:**
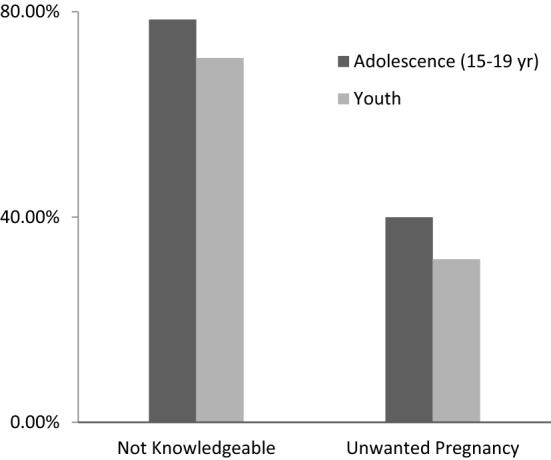
Comparison of incorrect knowledge of ovulation and unintentional pregnancy between adolescent and youth (Pearson’s Chi-square are significant, *p* < 0.05) (29 sub-Saharan African countries, 2008–2017)

Stratification analysis by age (Fig. 1) shows significant differences between adolescents (15–19) and emerging youths (20–24) who have incorrect knowledge of ovulation ($\chi^{2}_{(1)}$ = 1200, *p* < 0.001); and reported unintentional pregnancy ($\chi^{2}_{(1)}$ = 445.31, *p* < 0.001). Figure 1 shows that adolescent women (15–19) who had unintentional pregnancy were 39.95% and those with incorrect knowledge of ovulation were 78.47%. Furthermore, *t*-test analyses confirm that those with intended pregnancies are significantly older (*M *= 20.866; SD = 2.236) than those reported at least one unintentional pregnancy or child (*M *= 20.489; SD = 2.377; *t*_(73,234)_ = 21.202, *p *< 0.001). Similarly, those with correct knowledge of ovulation phase were significantly older (*M *= 19.707; SD = 2.712) than those without (*M *= 19.008; SD = 2.837; *t*_(160537)_ = 43.18, *p *< 0.001).

### Knowledge of ovulation, fertility intention, and contraceptive use

Table 2 presents the summary distribution of the explanatory and the outcome variables stratified by knowledge of ovulation. The prevalence of incorrect knowledge of ovulation was high among rural young women (age = 15–19), single, nulliparity, contraceptive users, those who desire to be pregnant, and not pregnant. Specifically, the prevalence of incorrect understanding of ovulation among pregnant women was 9.4% and 9.8% among those who were not pregnant ($\chi^{2}_{(1)}$ = 5.52, *p* = 0.019). Furthermore, we explore the association of knowledge of ovulation and the pattern of contraception use. Our analysis shows that 78.1% of women who reported not using any contraception did not have correct knowledge of the ovulation phase. On the other hand, 70% of users did not have the proper knowledge of ovulation. It is interesting to note that those women who had correct knowledge of the ovulation phase (30%) tend to use contraception more than those who did not (21.9%) ($\chi^{2}_{(4)}$ = 1500, *p* < 0.001 Table not shown). When those who reported current pregnancy were excluded, 80.2% did not use contraception, and it varies significantly among the selected countries. The prevalence ranges from 0.5% in Sao Tome to 9.1% in Nigeria (Table not shown). Additionally, regarding the pattern of contraceptive use based on pregnancy status, 70% of women who were pregnant at the time of the survey were not using any contraception, 7.6% used before pregnancy, 21.3% used since their last birth and of course, no woman who was pregnant was on contraception. Among those who were not pregnant, only 20% were currently using, 6.7% used since their last birth, 4.1% used before previous birth, and 68.9% never used contraception (Table not shown).

**Table 2:** Chi-square distribution of variables by knowledge of ovulation among women age 15–24 in 29 Sub-saharan African countries between 2008 and 2017

	Knowledge of ovulation		Total	*χ*^2^ (*p*-value)
	Knowledgeable (%)	Not knowledgeable (%)		
Age in 5-year groups				1154.513 (< 0.001)
15–19	18,585 (46.3)	72,730 (56)	91,315 (53.7)	
20–24	21,521 (53.7)	57,103 (44)	78,624 (46.3)	
Total (column)	40,106 (100)	129,833(100)	169,939 (100)	
Marital status				28.571 (< 0.001)
Never in union	22,472 (56)	74,455 (57.3)	96,927 (57)	
Currently in union	16,130 (40.2)	50,298 (38.7)	66,428 (39.1)	
Not currently in union	1503 (3.7)	5080 (3.9)	6583 (3.9)	
Total (column)	40,105 (100)	129,833 (100)	169,938 (100)	
Fertility status				45.669 (< 0.001)
No live birth	23,077 (57.5)	75,564 (58.2)	98,641 (58)	
One child	9458 (23.6)	28,806 (22.2)	38,264 (22.5)	
Two children	4928 (12.3)	16,204 (12.5)	21,132 (12.4)	
Three children	1961 (4.9)	6706 (5.2)	8667 (5.1)	
≥ 4 children	682 (1.7)	2553 (2)	3235 (1.9)	
Total (column)	40,106 (100)	129,833 (100)	169,939 (100)	
Place of residence				1588.663 (< 0.001)
Urban	19,197 (47.9)	47,702 (36.7)	66,899 (39.4)	
Rural	20,909 (52.1)	82,131 (63.3)	103,040 (60.6)	
Total (column)	40,106 (100)	129,833 (100)	169,939 (100)	
Contraceptive				934.705 (< 0.001)
User	30,844 (76.9)	108,556 (83.6)	139,400 (82)	
Nonuser	9262 (23.1)	21,277 (16.4)	30,539 (18)	
Total (column)	40,106 (100)	129,833 (100)	169,939 (100)	
Pattern of use				1500.247 (< 0.001)
Currently using	8489 (23.4)	19,775 (16.6)	28,264 (18.1)	
Used since last birth	1955 (5.4)	10,642 (8.9)	12,597 (8.1)	
Used before last birth	2103 (5.8)	4773 (4)	6876 (4.4)	
Never used	23,571 (65)	83,957 (70.3)	107,528 (69)	
Missed	163 (0.4)	298 (0.2)	461 (0.3)	
Total (column)	36,281 (100)	119,445 (100)	155,726 (100)	
Pregnancy desire				4.538 (0.033)
Yes	11,084 (68.3)	33,484 (67.4)	44,568 (67.6)	
No	5142 (31.7)	16,189 (32.6)	21,331 (32.4)	
Total (column)	16,226 (100)	49,673 (100)	65,899 (100)	
Currently pregnant				
No/not sure	36,195 (90.2)	117,682 (90.6)	153,877 (90.5)	5.52 (0.019)
Yes	3911 (9.8)	12,151 (9.4)	16,062 (9.5)	
Total (column)	40,106 (100)	129,833 (100)	169,939 (100)	

### Multivariate logistic regression

Table 3, model 1, includes all variables except knowledge of ovulation. Model 2 shows that compared to those who were knowledgeable about ovulation timing, women who were not knowledgeable had 15% higher odds of having an unintentional child (Wald = 6.14, *p* < 0.001); and women who did use contraception had 48% higher odds unintentional child (Wald = 18.29, *p* < 0.001). For unintentional pregnancy outcome in Table 3 model 4, those who had incorrect knowledge of ovulation were 17% more likely to report unintentional pregnancy when we controlled for other variables (Wald = 7.73, *p* < 0.001; model 4 in Table 3). Those who were married at the time of the survey had lower odds of unintentional child/pregnancy than those who were previously married, but the odds were higher for never-married women for both unintentional child and pregnancy.

**Table 3:** Non-stratified logistic regression for predicting unwanted pregnancy among women age 15–24 in 29 SSA countries between 2008 and 2017

Variable	Unwanted child(ren)				Unwanted pregnancy			
	Model 1		Model 2		Model 3		Model 4	
	Coef.	OR	Coef.	OR	Coef.	OR	Coef.	OR
	*n*1		*n*2		*n*3		*n*4	
Lack of knowledge of ovulation	–	–	0.14*	1.15	–	–	0.15*	1.17
Contraceptive users	0.39*	1.48	0.39*	1.48	–	–	–	–
Age	− 0.09*	0.92	− 0.09*	0.92	− 0.08*	0.93	− 0.08*	0.93
Currently married	− 0.74*	0.48	− 0.74*	0.48	− 0.69*	0.50	− 0.69*	0.50
Never married	0.95*	2.58	0.95*	2.57	0.93*	2.54	0.93*	2.54
Not currently married	1	1	1	1	1	1	1	1
Fertility	0.26*	1.30	0.26*	1.30	0.25*	1.28	0.25*	1.28
No education	− 1.08*	0.34	− 1.11*	0.33	− 1.09*	0.34	− 1.12*	0.33
Primary education	− 0.11	0.9	− 0.14	0.87	− 0.04	0.96	− 0.07	0.94
Secondary education	0.18*	1.19	0.16*	1.18	0.24*	1.27	0.22*	1.25
Rural residence	− 0.12*	0.89	− 0.13*	0.88	− 0.12*	0.89	− 0.12*	0.88
*Y*-intercept	1.25*		1.15		1.17		1.06	
McFadden’s *R*^2^	0.13		0.13		0.12		0.12	
Chi-square	9610.98		9649		11,313.51		11,373.69	
*N*	56,382		56,382		71,562		71,562	

*n*1–*n*4: serial analyses for all the models in STATA

*p < 0.05

### Stratified logistic models by marital status

To investigate the effect of marital status in the primary analysis in Table 3, we further disaggregated our data and analyzed it by marital status. In Table 4, among married cohorts (Sublevel A), the odds of contraceptive use were 1.58 for unintentional child (models 1 and 2), and the odds of incorrect knowledge of ovulation for both outcomes (i.e., unintentional child and pregnancy) were 1.20 and 1.21, respectively (models 2 and 4, Table 4). Incorrect knowledge of ovulation was insignificant for both unintentional child and pregnancy, but contraceptive use was positively associated with an unintentional child among the never-married and previously married cohorts.

**Table 4:** Multivariate logistic regression of predictors of unwanted children and pregnancy stratified by marital status among women age 15–24 in 29 Sub-saharan African countries between 2008 and 2017

	Unwanted child				Unwanted pregnancy			
	Model 1		Model 2		Model 3		Model 4	
	Coef.	OR	Coef.	OR	Coef.	OR	Coef.	OR
Sublevel A: Married	*n*5		*n*6		*n*7		*n*8	
Lack of knowledge of ovulation	–	–	0.18*	1.20	–	–	0.19*	1.21
Contraception	0.46*	1.58	0.46*	1.58	–	–	–	–
Age	− 0.08*	0.92	− 0.08*	0.92	− 0.07	0.94	− 0.06*	0.94
Fertility	0.33*	1.38	0.32*	1.38	0.29	1.34	0.29*	1.34
No education	− 1.04*	0.35	− 1.08*	0.34	− 1.05	0.35	− 1.08*	0.34
Primary education	− 0.04	0.96	− 0.07	0.93	0.06	1.06	0.02	1.02
Secondary education	0.23*	1.26	0.21*	1.24	0.32	1.37	0.30*	1.35
Rural	− 0.17*	0.85	− 0.18*	0.84	− 0.15	0.86	− 0.15*	0.86
*Y*-intercept	0.27		0.15		0.09		− 0.04	
Sublevel B: Never married	*n*9		*n*10		*n*11		*n*12	
Lack of knowledge of ovulation	–	–	0.03	1.03	–	–	0.06	1.06
Contraception	0.16*	1.18	0.16*	1.18	–	–	–	–
Age	− 0.09*	0.91	− 0.09*	0.91	− 0.11*	0.9	− 0.10*	0.9
Fertility	− 0.28*	0.75	− 0.29*	0.75	− 0.14*	0.87	− 0.14*	0.87
No education	− 0.94*	0.39	− 0.95*	0.39	− 1.01*	0.36	− 1.02*	0.36
Primary education	− 0.17	0.84	− 0.18	0.84	− 0.22	0.8	− 0.23	0.79
Secondary education	0.12	1.13	0.12	1.12	0.08	1.08	0.07	1.07
Rural	0.01	1.02	0.01	1.01	− 0.02	0.98	− 0.03	0.97
*Y*-intercept	2.96		0.03		3.16		3.12	
Sublevel C: Previously married	*n*13		*n*14		*n*15		*n*16	
Lack of knowledge of ovulation	–	–	0.11	1.12	–	–	0.11	1.12
Contraception users	0.40*	1.5	0.40*	1.50	–	–	–	–
Age	− 0.08*	0.92	− 0.08*	0.92	− 0.08*	0.92	− 0.08*	0.92
Fertility	0.17*	1.18	0.16*	1.18	0.15*	1.16	0.15*	1.16
No education	− 1.30*	0.27	− 1.33*	0.26	− 1.21*	0.3	− 1.25*	0.29
Primary education	− 0.38	0.69	− 0.41	0.66	− 0.26	0.77	− 0.29	0.75
Secondary education	0.06	1.06	0.04	1.04	0.15	1.16	0.13	1.14
Rural	− 0.15*	0.86	− 0.16*	0.85	− 0.16*	0.85	− 0.17*	0.84
*Y*-intercept	1.53		1.48		1.56		1.5	

*n*5–*n*16: serial analyses for all the models in STATA

*p < 0.05

In all models, increasing age reduces the odds of unintentional pregnancy or child irrespective of marital status. Parity or number of children increases the likelihood of unintentional pregnancy or child for both currently married and previously married but not for never-married young women. Unexpectedly, women with no education or primary education and who lived in rural areas were less likely to have unintentional pregnancy or child compared to those with higher education and lived in non-rural areas.

## Discussion

In this study, we examined the prevalence of knowledge of ovulation, contraception, and unintentional pregnancy among young women age 15–24 in 29 countries of SSA. We observed significant differences in the correct knowledge of the ovulation period based on age, marital status, and contraceptive use. The range of prevalence of unintended pregnancy found among the young population across the country (9–59%) is higher than those reported for all women (15–49 years) in 29 African countries (10.8–54.5%) by Ameyaw et al. (2019). Also, the rate of correct knowledge of ovulation (10.4–49%) among young African women (15–24 years) in this study is signifcantly lower than the general women population aged 15 and 49 (10.61–63.75%) in SSA (Iyanda 2020, *forthcoming*). The discrepancy in the rate of unintentional pregnancy and incorrect knowledge of the ovulation period is a function of the varying cultural and geographical background in SSA. Because our pooled data consist of heterogeneous ethnic groups from different countries, their approach to reproductive health beliefs and practices are expected to differ considerably (Garenne and Zwang 2006). For instance, female adolescents often experience pressure from several ends at the point of negotiation of sexuality and contraceptive use in South Africa. Therefore, they cannot take unilateral decisions on pregnancy prevention and contraceptive use (Wood and Jewkes 2006). Besides these beliefs, the political context within which unintended pregnancies are situated cannot be glossed over. According to Ameyaw et al. (2019), the lack of political commitment is a significant factor in many African countries where government attention has moved away from developmental challenges due to poor governance. Again, the intracontinental variations can be attributed to poor spousal communication, poor SES, lack of access to contraceptives in the rural area, and poor modern contraceptive knowledge. Consequently, these variations may have adverse implications for the sexual and reproductive health outcomes and on the childbearing age of certain groups, particularly for adolescents and youths in Africa.

Incorrect knowledge of ovulation was a significant predictor of unintentional child and pregnancy among women in SSA. Our analyses showed that most people who became pregnant were not aware of their ovulation period. These findings also show the deficit in a proper understanding of women’s reproductive health, which has implications for risky health behaviors, including premarital sexual practices leading to premarital sexual fertility among young women in Africa (Clark et al. 2017). Mainly, married young women who do not have the correct knowledge of ovulation were more likely to have an unintentional child and pregnancy compared with never-married or previously married women.

We had an overly different perspective in our data after we performed a robustness check by stratifying our data by marital status. Specifically, we found that high parity, secondary education, and use of contraception had significant positive associations with unintentional children among young married women. The same variables and lack of knowledge of ovulation had positive associations with an unwanted pregnancy. Meanwhile, increasing age significantly reduces the risk of unwanted pregnancy/child while high parity is a risk factor among currently and previously married.

The sociocultural contexts in which women reside are essential to the definition, discussion, and prevention of unintentional pregnancy. Some of these contextual factors include low education, low socioeconomic status, gender imbalances, inadequate laws prohibiting child marriage, and women’s rights (Hof and Richters 1999; Maswikwa et al. 2015; Iyanda et al. 2019). Previous studies have reported that women from poor households are more likely to report unintentional pregnancies due to early marriage as a means of escaping poverty (Handa et al. 1982). Among young married women, unwillingness to start a family at the time of pregnancy and poor knowledge of reproductive health, which includes the incorrect use of contraception, could be responsible for the prevalence of unintentional pregnancy (Santhya 2011).

Due to sociocultural factors such as religious affiliation and illiteracy, some women are not allowed to use contraception, which probably increases the likelihood of unwanted or unplanned pregnancy (Pinter et al. 2016; Prettner and Strulik 2017). In both stratified and non-stratified models, we found that the use of contraception increases the likelihood of unintentional child/pregnancy. These associations are counterintuitive and unexpected. It is used ideally to prevent unintentional pregnancy, which makes us suspect the effect of reverse causality. Though the effectiveness of contraception alone in reducing unintentional pregnancy/family planning is still not fully known due to cases of failures (Polis et al. 2016; Srinivasan and White 2019), there is no available data to support the observed positive association in this current study is due to contraceptive failures. Furthermore, it is tempting to argue that incorrect knowledge of ovulation among contraceptive users who might experience contraceptive failure may warrant the prevalence of unintentional pregnancy or child. Thus, it is plausible to argue that knowledge of ovulation and correct use of and access to contraceptives can form an effective strategy toward effective reduction of unintentional pregnancy, particularly among young women in developing countries.

**Fig. 2:**
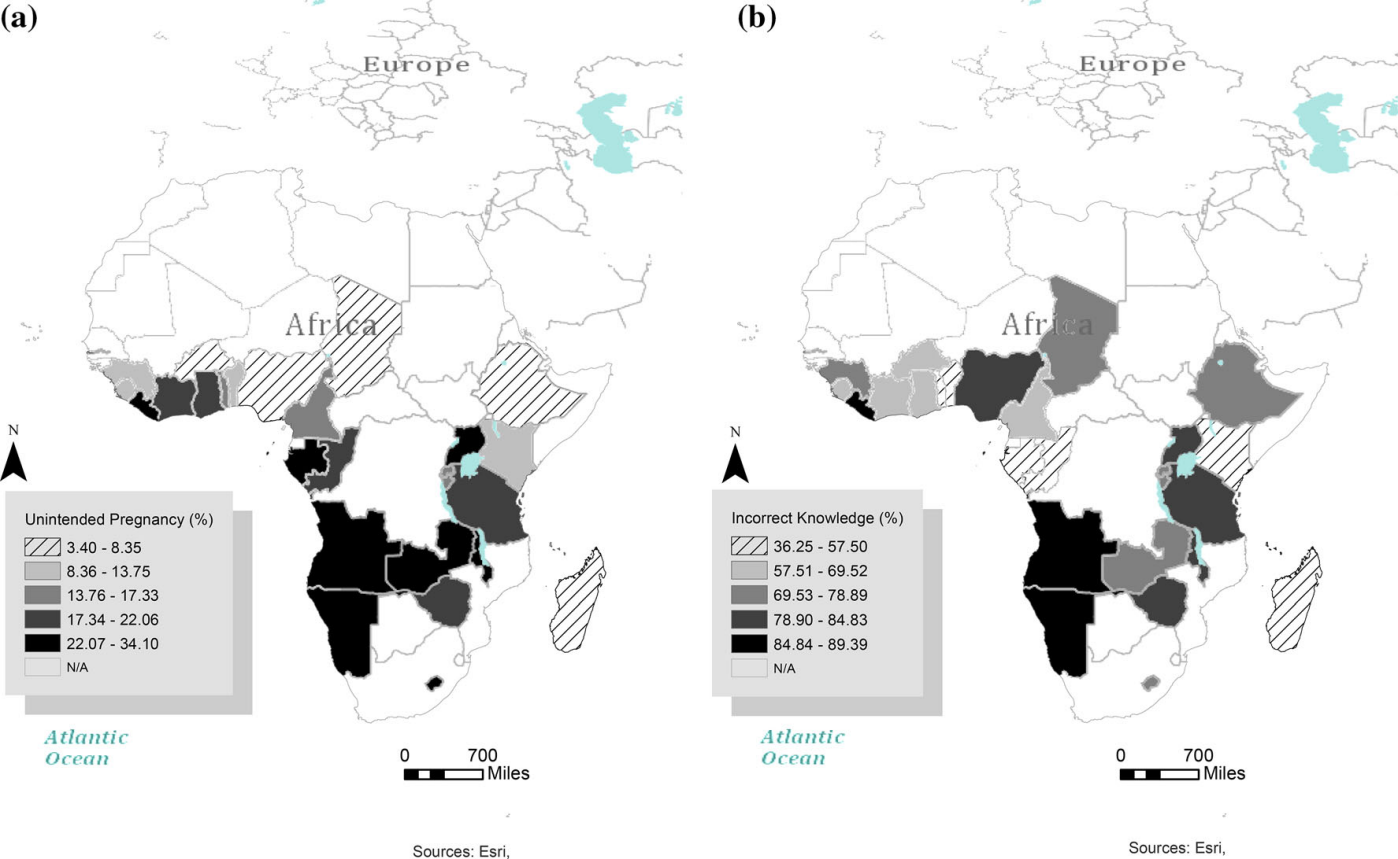
The prevalence of **a** unintentional pregnancy or fertility intention and **b** incorrect knowledge of ovulation (fertility awareness) among women age 15–24 in selected African countries (29 sub-Saharan African countries, 2008–2017)

Mapping knowledge of ovulation and unintended pregnancy helped us to visualize the spatial association between the two variables in countries in the southern region of Africa (Fig. 2). A plausible explanation for this is related to early marriage practices. In many African countries, early/child marriage is still extensively practiced, especially in predominantly Muslim communities, despite its harmful sociocultural outcomes (Maswikwa et al. 2015; Burton 2017). Sexual consent among countries in Africa varies and ranges from 13 years in Malawi to 18 years in some countries such as Burundi, Ethiopia, Uganda, and Rwanda, which is mostly influenced by the presence of working laws against child marriage (Maswikwa et al. 2015; Burton 2017). Married adolescents may not have appropriate knowledge of reproductive health (Sarkar et al. 2015), and they may be pressured to bear more at an early stage of their marriage (Cusick 1987; Bledsoe 1996). Furthermore, cultural views on abortion may contribute to the current prevalence of unintentional child/pregnancy in Africa (Burton 2017). Complicating this matter is the male manipulative schemes, uncontrolled self-gratification, and power relations of sexual decision making, which may contribute to unwanted pregnancy in SSA (Nalwadda et al. 2010; Bogale et al. 2011).

The repercussions of early/child marriage extend beyond unintentional pregnancy and include an effect on child mortality and developmental problems among children born from unintentional or unplanned pregnancies (Burton 2017) and also mental health issues among mothers themselves which this study did not cover.

The broad implication of this current study is that improved knowledge of ovulation needs to be emphasized among women of reproductive age. This can start from sex education at home, in the school curriculum, and during consultations with healthcare professionals. We believe that if women were more conscious of their ovulation timing, the rate of unintentional or unplanned pregnancies would reduce drastically. Poor knowledge of reproductive health is costlier in terms of psychological stress, unsafe abortion, maternal mortality, and children without enough resources to cater for them, which may eventually become a burden to the society. Pragmatic efforts, such as building community support for young women to discuss and share their experiences with professionals and educate them on fertility and sexuality, will be helpful.

It is imperative to mention that because the results were based on cross-sectional DHS data, our findings can only infer association, not causality. Admittedly, other factors may influence our analyzed data (DHS) as well as the interpretation. For instance, the responses given to questions on unintentional pregnancy may be affected by social desirability and response biases. Due to pregnancy difficulties, some women may have reported not wanting their pregnancy or child when they actually wanted it. This study did not account for the effect of diverse ethnic groups in different countries, which could also influence the knowledge of ovulation and contraceptive use. Hence, caution should be applied when interpreting these results due to the aggregated approach of this study.

As much as it would be desirable to control for some other relevant variables with the study outcomes, unintentional pregnancy/child, we could not extrapolate beyond our data. Some interesting variables were not available, which could have improved our understanding of the nature of unintentional pregnancy within the African context: (1) Data on those women who previously had cases of unintentional pregnancy that resulted in abortion, stillbirth, or miscarriage are lacking. This could be a follow-up on fertility intention questions. (2) Data on abortion due to unintentional pregnancy were not explicitly captured in DHS. (3) A measure of the economic or health costs of unintentional pregnancy among African women who reported unintentional pregnancies is unavailable. For instance, women who had experienced unintentional pregnancy have been reported to show symptoms of depression and exhibit risky behaviors such as suicide attempts, using unhealthy vulva objects, and eating concocted chemicals or herbals to abort the pregnancy (Yazdkhasti et al. 2015). Despite all these limitations, our approach paints a general picture of the subject matter addressed in this study.

### Conclusion

This study has improved the current understanding of the relationship between incorrect knowledge of ovulation and the prevalence of unintentional pregnancy among young women in Africa. We have shown that adolescent women have a poor understanding of ovulation and are more likely to report having an unintentional pregnancy. Generally, our non-stratified model showed that knowledge of ovulation is significantly associated with the prevalence of unintended pregnancy. However, there are variations when we stratified based on marital status, and it was interesting to observe such differences that could have been concealed without stratifying the data. Because we could not ascertain the role of contraception in reducing or preventing unintentional pregnancy, further research is needed to untangle the relationship between unintentional pregnancy/child and contraceptive use.

This study has important implications for improving women’s health education, particularly for unmarried young women in developing countries. Health providers in countries with a high prevalence of unintentional pregnancy and poor knowledge of ovulation should target this group (15–19), who are most likely to be at risk of unintentional pregnancy. Among married women within the age category, provisioning of in-depth knowledge of ovulation among their spouses will be the right approach in reducing unintentional pregnancy and unintentional child.
